# Effect of Smokeless Tobacco (Maras Powder) on the Epicardial Fat Thickness and Ventricular Repolarization Parameters

**DOI:** 10.3390/medicina59061127

**Published:** 2023-06-12

**Authors:** Kemal Göçer, Bayram Öztürk, Murat Kaniyolu, Mehmet Tekinalp

**Affiliations:** 1 Department of Cardiology, Faculty of Medicine, Kahramanmaras Sutcu Imam University, Kahramanmaras 46050, Turkey; 2 Department of Cardiology, Medical Park Goztepe Hospital, Istanbul 34730, Turkey; md.bayram12@gmail.com; 3 Department of Cardiology, Necip Fazıl City Hospital, Kahramanmaras 46080, Turkey

**Keywords:** smokeless tobacco, Maras powder, epicardial fat thickness, ventricular repolarization, Tp-e interval

## Abstract

*Background and Objectives*: Smokeless tobacco (ST) use has recently become an alternative to cigarettes, and it has been concluded that ST is at least as harmful as cigarettes. ST use is thought to play a role in the pathogenesis of arrhythmia by affecting ventricular repolarization. In this study, we aimed to examine the relationships of Maras powder (MP), one of the ST varieties, with epicardial fat thickness and new ventricular repolarization parameters, which have not previously been described. *Materials and Methods*: A total of 289 male individuals were included in this study between April 2022 and December 2022. Three groups, 97 MP users, 97 smokers, and 95 healthy (non-tobacco), were compared according to electrocardiographic and echocardiographic data. Electrocardiograms (ECG) were evaluated with a magnifying glass by two expert cardiologists at a speed of 50 m/s. Epicardial fat thickness (EFT) was measured by echocardiography in the parasternal short- and long-axis images. A model was created with variables that could affect epicardial fat thickness. *Results*: There were no differences between the groups regarding body mass index (*p* = 0.672) and age (*p* = 0.306). The low-density lipoprotein value was higher in the MP user group (*p* = 0.003). The QT interval was similar between groups. Tp-e (*p* = 0.022), cTp-e (*p* = 0.013), Tp-e/QT (*p* =0.005), and Tp-e/cQT (*p* = 0.012) were higher in the MP user group. While the Tp-e/QT ratio did not affect EFT, MP predicted the epicardial fat thickness (*p* < 0.001, B = 0.522, 95%CI 0.272–0.773). *Conclusions*: Maras powder may play a role in ventricular arrhythmia by affecting EFT and causing an increase in the Tp-e interval.

## 1. Introduction

Smokeless tobacco (ST) is widely used in some areas of the world, particularly attracting the attention of the young population [1]. Cigarette addicts may turn to ST to quit smoking due to its high nicotine content. Additionally, it can increase the probability of starting smoking in non-smokers. ST has been shown to affect many systems, especially the cardiovascular system [2]. Therefore, studies on ST have accelerated, and the effect of ST varieties on diseases has been investigated. Asian countries have an important place in terms of ST use. Maras powder (MP), a type of ST, is widely used in the southern region of Turkey. It is produced from the Nicotiana rustica L. plant. The leaves of this plant are ground into powder and mixed with wood ash. Generally, wood ash is obtained from oak or walnut wood. The MP is used not for chewing but by placing it between the lower lip and the jawbone. It is applied to the mucosa in amounts of about 1–2 g each time. This region allows nicotine to mix quickly into the blood since it is rich in the capillary bed. ST can be used many times a day in this way [3]. ST use, seen as harmless when compared to cigarettes, is increasing due to its easy accessibility and low price, and it is becoming a significant health problem worldwide [4].

Smokeless tobacco use has been shown to cause an increased cardiovascular risk similar to smoking. It is among the risk factors for hypertension, hypercholesterolemia, diabetes mellitus, atrial fibrillation, and metabolic syndrome [5]. In an electrocardiogram (ECG), the Tp-e interval, Tp-e/QT, and Tp-e/corrected QT are markers associated with ventricular repolarization that have been used to predict the development of arrhythmia in recent years. It has been reported that changes in these parameters cause ventricular arrhythmias and sudden death [6]. The MP causes a deterioration in echocardiographic parameters and changes in parameters showing ventricular repolarization in an ECG [7]. However, there have been no studies evaluating new ventricular repolarization parameters. In addition, the effect of MP on the epicardial fat thickness (EFT) between the myocardium and the visceral pericardium is unknown. The EFT is mediated by pathophysiological mechanisms similar to the obesity process, and its relationship with atherosclerotic diseases and rhythm disturbances has been shown [8]. It was thought that the absence of fascial structures separating myocardial and epicardial adipose tissue (EAT) might be responsible for the arrhythmia etiology and might be related to ventricular repolarization parameters [8,9]. In this study, we evaluated the relationship of MP use with new ventricular repolarization parameters in an ECG and its effect on EFT.

## 2. Materials and Methods

### 2.1. Study Design

There were a total of 289 male participants, of whom 97 used only MP and 97 were smokers. The remaining 95 were healthy (not using MP and cigarettes). Participants were included in the prospective study from three different centers in the same geographical region who applied to the cardiology outpatient clinic between April 2022 and December 2022. Frequency of use of MP, usage time, and usage method (direct contact use with oral mucosa or wrapped in cigarette paper) were noted. Those with history of coronary artery disease, arterial hypertension, diabetes mellitus, thyroid dysfunction, anemia, renal failure, lung disease, electrolyte imbalance, severe heart valve disease, left ventricular ejection fraction (LVEF) less than 50%, ST-T wave changes, atrioventricular conduction abnormalities, and bundle branch block on ECG, use of any drug that affects the cardiac conduction system, poor quality echocardiographic and electrocardiographic imaging were excluded from the study.

Coronary artery disease was excluded as follows: Those with no previous history of coronary angiography, those who were evaluated as having normal epicardial arteries after coronary angiography, according to the 2021 American guideline using diagnostic tests that exclude coronary artery disease (ECG, cardiac exercise stress test, and computer tomography coronary angiography) [10].

Written consent was obtained from all participants. In addition, the local committee obtained ethics committee approval.

### 2.2. Definitions of Tobacco Use

Smokers: Individuals who smoked at least one pack/year and smoked in the last week were considered. One pack of cigarettes was counted as 20 times use.

Maras powder users: Individuals who used MP for at least one pack/year and who had been using MP within the last week were included in this study. One packet of MP was considered equal to 20 times usage.

### 2.3. Electrocardiographic Parameters

Electrocardiography was taken at a speed of 50 m/s and an amplitude of 10 mm/mV at rest for all participants. The ECGs were evaluated by two specialist doctors with the help of a magnifying glass. The heart rate per minute was calculated by dividing 1500 mm by the R-R mm distance. The QT interval was measured from the beginning of the QRS complex to the end of the T wave. V5 derivation was considered in the measurement of the QT interval. When the measurement from V5 was insufficient, QT was determined by taking the average of V4–V5. QT intervals were calculated in all 12 leads for QT dispersion (QTd). The QTd was found by calculating the difference between the leads’ maximum and minimum QT. The Tp-e interval was defined as the distance between the peak of the T wave and the endpoint. The heart rate corrected QT interval (cQT) was calculated using Bazett’s formula. Tp-e/QT ratio and Tp-e/cQT ratio were found. The frontal QRS-T angle was the absolute difference between the QRS axis and T axis (frontal QRS-T angle = QRS axis − T axis). Information was obtained from the automatic report section of the surface ECG device. If this angle exceeded 180°, the current angle was subtracted from 360° and recalculated.

### 2.4. Echocardiographic Parameters

Echocardiography was performed on all participants according to American Society of Echocardiology (ASE) guidelines and European Society of Cardiovascular Imaging guidelines [11,12]. It was applied to individuals in the left lateral and decubitus position. Doppler echocardiography and m-mode parameters were measured in two dimensions. Left ventricular ejection fraction, interventricular septal thickness, posterior wall thickness, left ventricular diastolic dysfunction, and left ventricular systolic dysfunction values were noted. Diastolic mitral flow patterns were measured by pulse Doppler echocardiography. LVEF was calculated via Simpson’s biplane technique. Parasternal long-axis and short-axis imaging measurements of the EFT on the right ventricular free wall were used. The EFT was defined as a low-density area between the myocardium and the visceral pericardium. The EFT was measured perpendicular to the right ventricle at the systole’s end and three cardiac cycles. The Devereux formula calculated the left ventricular mass index (LVMI). The formula usually stated as 0.8 × {1.04 × [[LVEDD + IVSD + PWD]^3^ − LVEDD^3^]} + 0.6 g was derived by dividing body surface area and assuming LV dimensions in centimeters.

### 2.5. Statistical Analysis

All data were analyzed by SPSS 22 (SPSS Inc., Chicago, IL, USA) software. Numerical data were expressed as standard deviation or median according to their convenience. Independent Student’s *t*-test was used to compare the averages between two independent groups. One-way ANOVA test was performed to compare MP users, smokers, and control groups with numerical data. Bonferroni test was used for those who achieved homogeneity of variance in two comparisons and Tamhane’s T2 for those who did not. Correlation analysis was performed between EFT and ECG parameters. Multivariate linear regression analysis was performed to investigate the factors affecting EFT. The Tp-e/QT, body mass index (BMI), age, use of ST, and low-density lipoprotein were included in the regression model. Interobserver agreement of EFT and ECG data was calculated using the Bland–Altman analysis, and intraclass correlation coefficients were used to assess intra-observer agreement. *p* value < 0.05 was considered significant.

## 3. Results

The groups’ BMI (*p* = 0.672) and age (*p* = 0.306) were similar. However, in the biochemical parameters between the groups, the LDL (*p* = 0.003) value was significantly higher in the MP group. In post hoc analyses, no significant difference was observed between the smoking and control groups (*p* = 0.867). A higher tobacco pack/year use was observed in smokers than in MP users (*p* < 0.001). Other biochemical parameters are shown in Table 1. Electrocardiographic and echocardiographic data are analyzed in Table 2.

**Figure 1:**
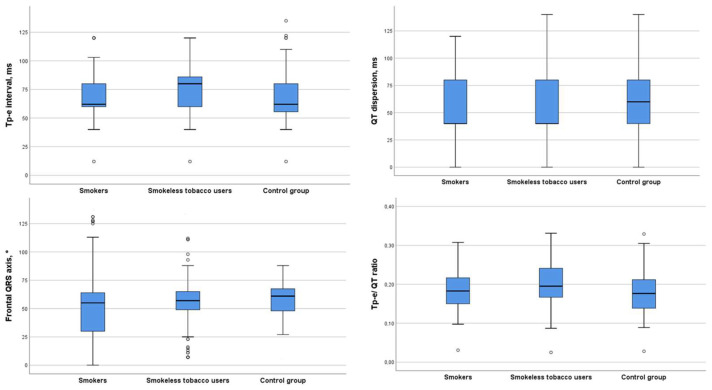
A boxplot graph showing variables between groups.

There was no difference between the groups in the QT interval and its derivatives (*p* > 0.05). In Figure 1, the ECG parameter values of the three groups are shown as boxplots. The Tp-E interval (*p* = 0.022) and epicardial fat thickness (*p* < 0.001) were significantly higher in the MP group.

**Figure 2:**
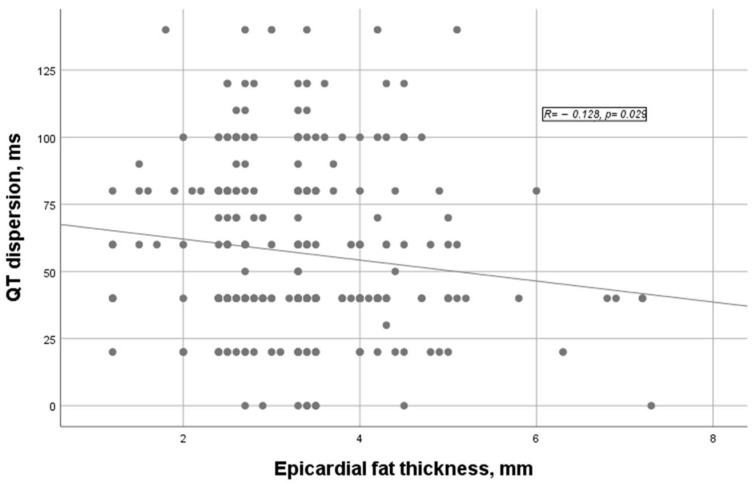
A correlation plot of epicardial fat thickness and QT dispersion.

The correlation of EFT with parameters on the ECG was weak or insignificant. (Tp-e, *p* = 0.426, R = 0.047; Tp-e/QT ratio, *p* = 0.043, R = 0.119; cQT, *p* = 0.789, R = −0.016). As shown in Figure 2, QTd was negative and weakly correlated (*p* = 0.029, R = −0.128).

Dummy variables were created to examine the effect of MP use on epicardial fat thickness, and linear multivariate regression analysis was applied. MP was found to be an independent precursor of the epicardial fat thickness (*p* < 0.001, B = 0.522, 95%CI 0.272–0.773) (Table 3).

## 4. Discussion

Tobacco is an addictive agent widely used worldwide that increases dopamine levels in the central nervous system thanks to the nicotine it contains [13]. It is obtained from the leaves of Nicotiana tabacum and Nicotiana rustica plants. N. tabacum and N. rustica are similar in terms of alkaloid content. However, nicotine is 4–5 times higher in N. rustica [14]. Tobacco is divided into two types, smoky and smokeless. Recently, smoking has been declining since smoking affects many organs and systems. This has created a worldwide anti-smoking public opinion. However, there is an increase in the use of ST, especially among young people. The belief that it is less harmful than smoking, its relatively easy accessibility, and the high amount of nicotine has led to a trend in ST [1]. The association of chemicals and nicotine in MP with many diseases suggests it is as harmful as cigarettes [15]. Many types of ST are used worldwide. The MP frequently used in the southeast region of Turkey is a type of ST obtained from the N. rustica plant and placed between the cheek mucosa and teeth. MP is associated with oral mucosal, cancer, and cardiovascular diseases [5]. In addition, it is thought that MP may cause malignant arrhythmias and sudden death, similar to some studies conducted with smoking. Changes in the distribution of transmural repolarization may play a role in this process [16]. This study aimed to show the relationship between MP use and new ventricular repolarization parameters and EFT, which have not been reported before. In this study, the use of MP, a type of ST, showed that it may be a factor in increased EFT. In addition, Tp-e-related parameters were elevated in those using MP. In this respect, this study can be helpful for future studies, as it shows that MP, commonly used in the endemic regions, is as harmful as smoking and can cause sudden cardiac death by triggering an arrhythmia.

The mechanism for most sudden cardiac deaths is re-entry ventricular arrhythmias [17]. The distribution of transmural repolarization between layers of the heart is responsible for these arrhythmias. Middle myocardial cells are called M cells. They have the most extended action potential, while epicardial cells have a relatively shorter ventricular repolarization time. The repolarization of epicardial cells in the ECG coincides with the peak of the T wave, while the repolarization of M cells coincides with the end of the T wave. This interval is called the Tp-e interval. Prolongation of the Tp-e interval has been associated with ventricular arrhythmias and increased sudden cardiac death [16]. In this study, the Tp-e interval was higher in the MP group. The effects of chemicals in MP and its high nicotine content compared to cigarettes may cause the development of ventricular arrhythmia and an increased risk of sudden cardiac death. These features may be more dangerous in the presence of underlying idiopathic ventricular arrhythmias or disturbances in the electrolyte balance in the blood. The Tp-e interval can be affected by factors such as body weight and heart rate. Therefore, the Tp-e/QT and Tp-e/cQT ratios can be better indicators [18]. Gupta P et al. reported that the Tp-e/QT ratio could indicate ventricular arrhythmia in patients with long QT syndrome, Brugada syndrome, and obstructive cardiomyopathy [6]. Yayla Ç et al. found a reduced Tp-e/QT ratio after successful radiofrequency ablation in patients with idiopathic ventricular arrhythmias [19]. They showed that Tp-e/QT might play a role in the pathophysiology of idiopathic ventricular arrhythmias. In our study, the Tp-e/QT ratio was higher in MP users than in the smoking and control groups. However, we can say that this difference is due to the change in the Tp-e interval.

The QT interval, cQT, and QT distribution of ECG parameters are associated with cardiovascular diseases (left ventricular hypertrophy, acute coronary syndrome, heart valve prolapses) and systemic diseases such as rheumatoid arthritis and systemic sclerosis. The QTd is calculated by subtracting the maximum and minimum QT values. QTd values are controversial, and the normal range varies across studies. Those with QTd > 60 ms had twice the risk of sudden cardiac death than those with <30 ms. Several studies have shown that they are more reliable measures of transmural distribution than Tp-e/QT ratios [20]. In our study, no difference was found between the groups regarding QTd.

The frontal QRS and T wave axes are routinely reported on most current ECG machines. The frontal QRS-T, a ventricular repolarization variable, is the absolute value of the difference between the QRS axis and the T wave axis. Studies show that the QRS-T angle is more robust and reproducible in clinical use compared to the QT interval. The QRS-T angle can be calculated using the QRS-T angle in the spatial and frontal planes [21]. QRS-T angle measurement with the spatial method cannot be measured routinely from surface ECG and requires special computer programs [22]. On the other hand, the frontal QRS-T angle can be easily detected from the surface ECG (by subtracting the QRS axis from the T axis) because many ECG devices have a QRS axis and T axis in the automatic report part [23]. The frontal QRS axis did not differ in the three groups in this study. Previous studies have shown a significant correlation between the spatial QRS-T angle and the left ventricular mass [24]. The fact that the groups’ LVMI was similar may have caused no difference in the frontal QRS-T angle.

Smoking is known to affect cholesterol levels. It causes an increase in total cholesterol, LDL cholesterol, and a decrease in HDL cholesterol. An increase in cholesterol levels may predispose to arrhythmia [25]. The anti-arrhythmic effect of statin therapy used in treating hypercholesterolemia or in cases of vascular events is named a pleiotropic effect [26]. Hypercholesterolemia causes changes in ventricular repolarization parameters. After using statin therapy in patients with hypercholesterolemia, ECG showed a decrease in Tp-e and QT intervals compared to before. QTd can be affected by the circadian rhythm. QT fluctuation was highest in the morning hours [27]. It explains why ventricular fibrillation and sudden death are more common in the morning. Atorvastatin drug use has shown that it reduces QTd and may be correlated with a reduction in the risk of sudden death [28]. Our study found no difference between the groups in lipid values other than LDL. Therefore, initiation of early statin therapy or discontinuation of ST use due to possible side effects may be considered in the presence of higher LDL cholesterol in MP users.

Inflammatory parameters that play a role in the atherosclerotic process have been well-defined before. It has been stated that smoking is also a mediator of the atherosclerotic process and may cause an increase in EFT [29]. In an investigation, EFT was found to be 3.8 mm in smokers and 2.6 mm in non-smokers. Another study showed that epicardial fat thickness over 2.5 mm could predict atherosclerosis [30]. In our study, EFT was found to be 3.70 mm, 3.07 mm, and 3.15 mm in the MP user, control group, and smokers, respectively. The EFT may increase with age. However, the fact that our study group consisted of a relatively young population does not explain this situation. Our view is based on the high BMI, which is a sign of obesity in the participants in this research. The longer the tobacco is consumed, the higher the risk of inflammation and atherosclerosis. Rom et al. identified high blood c-reactive protein levels and increased EFT in smokers. The same study showed a poor correlation between pack/year duration and EFT in smokers [31]. The duration of smoking was greater than the duration of ST use, but EFT was higher in ST users. This may be a result of the high levels of nicotine in MP, compared with cigarettes. In addition, MP may play a more aggressive role in the inflammatory process, with a mechanism that we do not know yet. Our hypothesis can be supported by clarifying the pathophysiology with future studies.

Epicardial adipose tissue has properties similar to intra-abdominal adipose tissue. The correlation of an increase in EAT with many systemic diseases has been reported. The EAT is directly connected to the myocardium. In addition to its protective effects on the heart, it also has harmful effects. The EAT prevents the heart from being exposed to large amounts of fatty acids [32]. In a pathological condition, it plays a role in the inflammatory process, releasing markers that can damage the coronary arteries and myocardium [33]. The EFT was found to be a risk factor for coronary artery disease in patients with DM. Al-Alousi LM. reported that EFT was positively correlated with LVMI independent of coronary artery disease in 117 autopsy examinations [34]. The relationship between left atrium enlargement and EFT has been demonstrated. Moreover, it has been reported to play a role in developing arrhythmias. Studies evaluating EFT with ventricular repolarization parameters are limited. A relationship has been shown between increased Tp-e and EFT. However, studies on cQT are conflicting, showing a positive and negative correlation [35]. It has been hypothesized that EFT causes repolarization heterogeneity, triggering a one-way block after the extra beat and initiating the re-entry circuit. In addition, EFT causes a slowdown in myocardial infarction-like activation time. Thus, this can initiate ventricular arrhythmias by forming a re-entry circuit [8]. In our study, EFT was higher in MP, although smoking was higher on a pack/year basis. The MP was found to affect EFT. Considering that BMI and LDL variables increase in EFT according to previous studies, the effect of MP was significantly higher.

This study has some limitations. First, the effect of the participants’ emotional states on the ECG parameters is not precisely known. The second is that since MP is not a fabricated product, its mixing ratios vary slightly. Third, the use of MP is almost non-existent in women, so the study was conducted with male individuals.

## 5. Conclusions

In conclusion, MP may play a role in ventricular arrhythmia by affecting EFT and causing an increase in the Tp-e interval.

## Figures and Tables

**Table 1:** A comparison of demographic and laboratory values of the groups.

	Smokers *n* = (97)	Smokeless Tobacco Users *n* = (97)	Control Group *n* = (95)	* *p*
Age	33.32 ± 8.29	35.16 ± 8.74	34.56 ± 8.43	0.306
BMI	25.22 ± 3.63	25.50 ± 4.33	25.01 ± 3.44	0.672
Time of usage, pack/year	6.00–7.00	3.00–2.00	0	^a^ **<0.001**
Glucose, mg/dL	101.48 ± 17.07	103.45 ± 16.81	102.41 ± 17.17	0.727
Total cholesterol, mg/dL	197.93 ± 55.48	194.78 ± 56.65	186.95 ± 46.32	0.339
TG, mg/dL	182.52 ± 116.36	178.36 ± 96.21	176.42 ± 101.87	0.919
HDL, mg/dL	46.56 ± 13.56	44.95 ± 12.39	45.31 ± 12.83	0.476
LDL, mg/dL	110.23 ± 35.28	127.36 ± 39.38	113.80 ± 36.07	**0.003**
WBC, 10^3^/µL	7.44 ± 1.83	7.13 ± 1.91	7.37 ± 1.78	0.464
Hemoglobin, g/dL	14.78 ± 1.41	14.85 ± 1.38	15.23 ± 1.34	0.056
Platelet, 10^3^/µL	281.50 ± 76.16	285.31 ± 79.18	279.35 ± 82.42	0.870
Sodium, mEq/L	139.64 ± 3.09	139.62 ± 3.10	139.64 ± 3.06	0.999
Potassium, mEq/L	4.55 ± 0.43	4.51 ± 0.40	4.50 ±0.46	0.697
Magnesium, mg/dL	2.09 ± 0.17	2.04 ± 0.16	2.08 ± 0.19	0.125
Calcium, mg/dL	9.37 ± 0.37	9.44 ± 0.36	9.45 ± 0.40	0.297

*p* < 0.05 are shown in bold. * Analysis of variance and Kruskal–Wallis test. ^a^ Independent samples *t*-test. BMI, body mass index; HDL, high-density lipoprotein; LDL, low-density lipoprotein; TG, triglyceride; WBC, white blood cell.

**Table 2:** A comparison between electrocardiographic and echocardiographic parameters of groups.

	Smokers *n* = (97)	Smokeless Tobacco Users *n* = (97)	Control Group *n* = (95)	* *p*
Electrocardiographic parameters				
Heart rate, bpm	79.88 ± 13.53	81.79 ± 16.30	79.99 ± 15.17	0.610
QT interval, ms	377.53 ± 51.51	377.89 ± 51.99	379.84 ± 48.80	0.944
Maximum QT interval, ms	391.54 ± 64.65	390.92 ± 64.74	398.21 ± 64.62	0.689
Minimum QT interval, ms	338.14 ± 53.37	332.78 ± 42.44	338.73 ± 50.80	0.650
QTd, ms	53.40 ± 27.53	57.93 ± 35.11	59.47 ± 29.40	0.366
cQT interval, ms	433.34 ± 55.06	437.37 ± 63.16	435.41 ± 59.17	0.894
Tp-e interval, ms	68.55 ± 18.94	74.46 ± 20.45	66.72 ± 21.19	**0.022**
cTp-e interval, ms	78.40 ± 21.03	86.19 ± 24.16	76.71 ± 25.82	**0.013**
Tp-e/QT ratio	0.18± 0.04	0.20 ± 0.05	0.17 ± 0.05	**0.005**
Tp-e/cQT ratio	0.15 ± 0.04	0.17 ± 0.05	0.15 ± 0.04	**0.012**
Frontal QRS-T angle, °	54.57 ± 31.40	56.78 ± 29.34	60.65 ± 34.05	0.404
Echocardiographic parameters				
LVEF, %	60.84 ± 3.67	60.81 ± 3.60	60.81 ± 3.91	0.997
LVDD, mm	47.19 ± 2.60	48.01 ± 2.65	47.87 ± 2.32	0.054
LVSD, mm	32.98 ± 2.17	32.87 ± 2.00	33.41 ± 2.20	0.176
IVST, mm	8.98 ± 1.26	8.96 ± 1.30	9.06 ± 1.27	0.837
PWT, mm	8.21 ± 1.29	8.26 ± 1.26	8.35 ± 1.23	0.737
Mitral E velocity, cm/sn	56.49 ± 8.39	55.93 ± 9.41	57.26 ± 9.10	0.587
Mitral A velocity, cm/sn	44.07 ± 7.15	44.23 ± 6.85	44.39 ± 7.19	0.704
Epicardial fat thickness, mm	3.15 ± 0.91	3.70 ± 1.13	3.07 ± 0.84	**<0.001**
LVMI (g/m^2^)	73.52 ± 17.64	74.32 ± 16.50	76.13 ± 16.44	0.550

*p* < 0.05 are shown in bold. * Analysis of variance and Kruskal–Wallis test. IVST, interventricular septal thickness; LVDD, left ventricular diastolic dysfunction; LVEF, left ventricular ejection fraction; LVSD, left ventricular systolic dysfunction; PWT, posterior wall thickness, QTd, QT dispersion.

**Table 3:** Analysis of variables that may affect the epicardial fat thickness.

	B	β	* *p*	95%Cl	
				Lower	Upper
Low-density lipoprotein	0.002	0.073	0.218	−0.001	0.005
Use of Maras powder	0.522	0.244	**<** **0.001**	0.272	0.773
Tp-e/QT ratio	1.403	0.074	0.207	−0.782	3.589
Body mass index	−0.002	0.015	0.909	−0.032	0.028
Age	0.003	0.022	0.708	−0.011	0.016

*p* < 0.05 are shown in bold. * Multivariate regression analysis.

## Data Availability

All data generated or analyzed during this study are included in this article. Further inquiries can be directed to the corresponding author.
